# THE RELATIONSHIP BETWEEN CAREGIVING TRANSITION AND SUBJECTIVE AGE: THE ROLE OF SENSE OF CONTROL

**DOI:** 10.1093/geroni/igae098.3281

**Published:** 2024-12-31

**Authors:** Hyojin Choi, Kristin Litzelman

**Affiliations:** University of Vermont, Vermont, United States; University of Wisconsin Madison, Madison, Wisconsin, United States

## Abstract

Subjective age (SA) – how old one feels in relation to their chronological age – varies considerably in challenging situations. The strain and demands of family caregiving might therefore influence patterns of SA. Furthermore, psychological resources that enable family caregivers to deal with stressors, such as sense of control, may buffer these adverse effects. The purpose of this study was to examine the relationship between transitions in caregiving status and SA, and the moderation role of sense of control (perceived constraint and mastery) in this relationship. Using longitudinal data from the Health and Retirement Study (2006–2020), multilevel analyses were conducted to assess both within- and between-person relationships among caregiving transition, sense of control and subjective age. The sample consisted of married/partnered respondents (n=14,944 observations), and we categorized them into five caregiving transition groups: new caregivers, previous caregivers, long-term caregivers, potential caregivers, and non-caregivers based on caregiving status for their spouse/partner with limitations on daily living activities in consecutive waves. The findings show that caregiving transition interacted with perceived constraints and mastery to correlate with SA. Namely, among new caregivers, higher average levels of perceived constraints were associated with older SA (Beta=1.36, p=0.017). Among previous caregivers, higher within-person levels of perceived mastery were associated with younger SA (Beta=-2.66, p=0.001). These findings highlight the importance of developing and implementing interventions tailored to support spouses across the caregiving trajectory in having healthy levels of psychological resources, such as skills training programs and psychoeducation.

